# From linear to circular: the impact of economic policies and technological innovations on greenhouse gas emissions in the Netherlands

**DOI:** 10.1186/s13021-025-00297-1

**Published:** 2025-05-24

**Authors:** Qamar Abbas, Muhammad Imran, Abdul Sattar

**Affiliations:** 1 School of Business, Hengxing University Qingdao, Qingdao, China; 2 Bahria Business School, Bahria University Islamabad, Islamabad, Pakistan

**Keywords:** Circular economy, Greenhouse gases emissions, Technological innovation, Environmental Tax Policy, Wavelet coherence analysis

## Abstract

The Netherlands, recognized as a leader in promoting circular economy principles, is actively implementing laws, incentives, and public–private collaborations to reduce raw material consumption by minimizing extraction and encouraging sharing and reuse. Emphasizing the durability and extended use of materials and goods is crucial in this transition. This study investigates the long-term and causal impacts of circular economy practices, technological innovation, environmental tax policies, economic instability, and industrialization on greenhouse gas (GHG) emissions in the Netherlands, covering the period from the first quarter of 2010 to the fourth quarter of 2022. Employing advanced econometric techniques, including the bounds test of co-integration, autoregressive distributed lag models, wavelet coherence analysis, and gradual shift causality tests, the study reveals that circular economy practices, technological advancements, and environmental taxation significantly reduce GHG emissions in both the short-run and the long-run. Conversely, economic instability and industrialization are found to contribute positively to GHG emissions. The wavelet coherence analysis further highlights the substantial interplay between GHG emissions and the independent variables studied. Based on these findings, the study underscores the need for the Netherlands to intensify efforts in reducing GHG emissions, curbing the use of virgin materials in construction, and investing in recycling technologies to advance its circular economy goals.

## Introduction

Each year, approximately 100 billion tons of resources are extracted and supplied to the global economy, encompassing a diverse range of materials such as metals, minerals, fossil fuels, and organic matter derived from plants and animals. However, according to the circulatory gap report (2020), only a mere 8.6% of these resources are repurposed or recycled [27]. This unsustainable and excessive consumption has severe consequences for human health, biodiversity, and the overall well-being of our planet. As a result, the urgency to transition from traditional linear economic models—characterized by the use-and-dispose paradigm—to a circular economy is increasingly evident [74].

The circular economy is designed with the primary objectives of minimizing waste and pollution, extending the lifecycle of resources and products, and allowing natural systems to regenerate [36]. A significant driver of the need for a circular economy is the escalating levels of GHG in the Earth’s atmosphere, which are already causing profound changes in our climate. Solid waste management practices contribute directly to GHG emissions, with incineration facilities releasing nitrous oxide and landfills generating methane as waste decomposes [20]. In this context, the circular economy presents an opportunity to optimize the use of products, materials, and natural resources while simultaneously transforming waste into valuable inputs and reducing overall waste production [47]. Moreover, in the face of an escalating ecological deficit, it is imperative that humanity expedite its efforts to achieve the Sustainable Development Goals (SDGs) to avert irreversible environmental degradation [34]. The urgency of this transition requires transformative actions across sectors, aligning economic, social, and environmental objectives to create a resilient, low-impact global framework that ensures long-term sustainability for future generations [19].

**Fig. 1:**
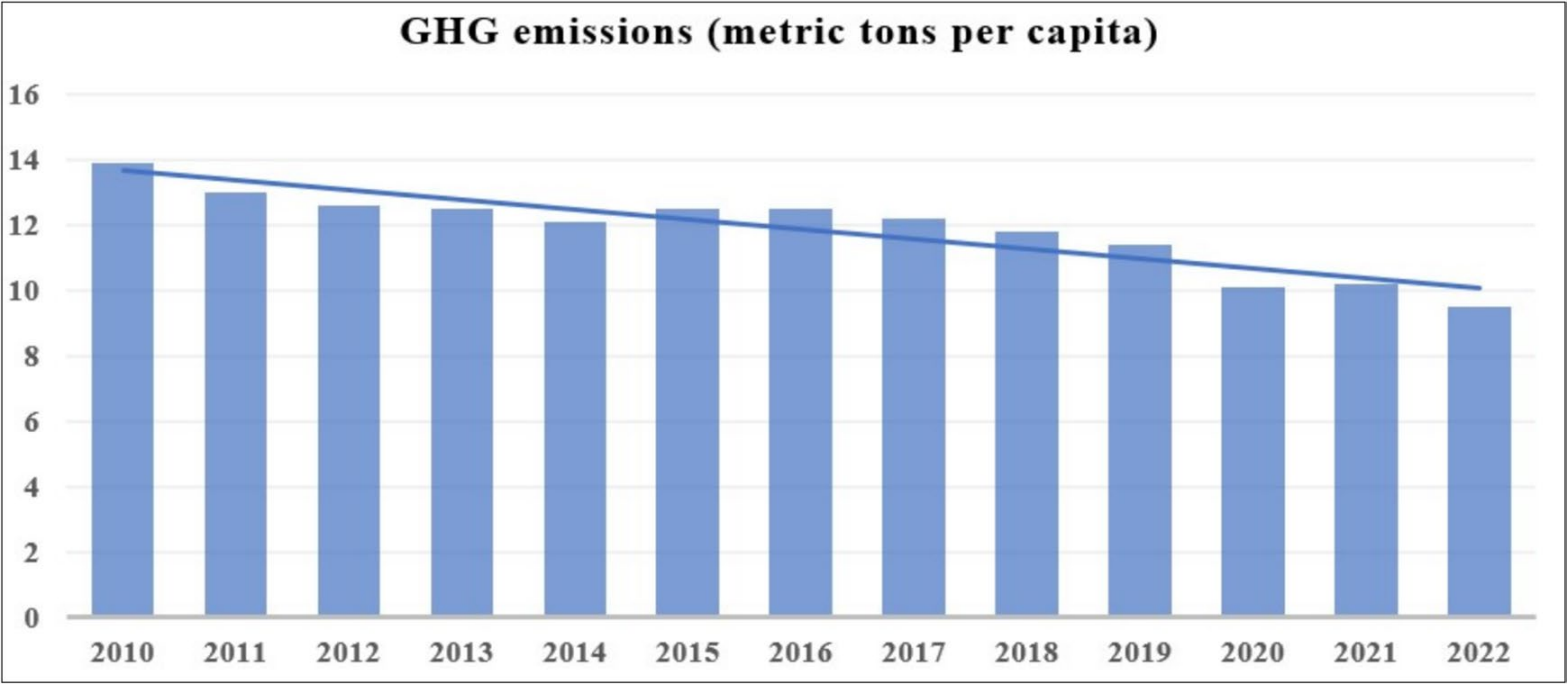
GHG emissions (metric tons per capita) in the Netherlands. Source: Eurostat (https://ec.europa.eu/eurostat)

The focus of this research is the Netherlands, a country selected due to its relatively high per capita GHG emissions compared to other European nations. In 2018, the Netherlands recorded per capita emissions of 11 tons of CO_2_, a figure that surpasses the European average by over 30% (Fig. 1). When compared to countries with similar per capita GDPs, such as Austria, Denmark, France, Italy, Norway, Sweden, and the United Kingdom (UK), whose emissions range from 5 to 8 tons, the Netherlands’ emissions are significantly higher [13]. Despite this, the Dutch economy has made strides in reducing its carbon intensity, achieving a 29% reduction between 2005 and 2019, albeit at a slightly slower pace than the European Union (EU) average. Notably, the energy sector in the Netherlands achieved a 15% reduction in emissions during this period, driven in part by policy measures such as the introduction of carbon pricing. The Netherlands supports the EU climate goals, which include reaching net-zero GHG emissions by 2050. By 2030, the EU "Fit for 55" package seeks to reduce GHG emissions by 55% from 1990 levels, which will have an impact on national policy.^1^

Recognizing the critical need to address its elevated GHG emissions, the Netherlands took a pioneering step in 2016 by launching the government-wide program ‘A Circular Economy in the Netherlands by 2050.’ This initiative not only demonstrated the country’s commitment to reducing emissions but also played a crucial role in raising public awareness and fostering a culture of sustainability. The Netherlands’ proactive approach aligns with the growing recognition of the circular economy as a viable strategy for achieving global climate goals, particularly the targets outlined in the Paris Agreement [95]. Moreover, the Netherlands has established extended producer responsibility (EPR) for a wide range of items, including packaging, electronics, and batteries. These systems hold manufacturers responsible for the recycling and reuse of items at the end of their lifespan [68].

The Netherlands has set ambitious targets to reduce GHG emissions through a combination of policy initiatives and legislative measures. By 2023, the country aims for at least 16% of its energy production to be derived from renewable sources. Furthermore, GHG emissions are projected to decrease by 49% by 2030 compared to 1990 levels, with a more aggressive target of reducing CO_2_ emissions by at least 55% by the same year. These milestones are part of a broader strategy to achieve a climate-neutral economy by 2050, culminating in a remarkable 95% reduction in GHG emissions from 1990 levels. These targets underscore the Netherlands’ commitment to mitigating climate change and transitioning toward a sustainable future (Ministry of Infrastructure and Water Management & Ministry of Economic Affairs, 2016).

A critical component of this strategy is the circular economy, which emphasizes the reuse and recycling of products and materials within the economy for as long as possible, thereby minimizing waste and reducing the demand for new resources [88]. In this study, the circular material use rate serves as a proxy for measuring the effectiveness and progress of the circular economy, following the methodologies of Hondroyiannis et al. [49] and Neves and Marques [74]. The circular economy fundamentally differs from the traditional linear economic model, which operates on a ‘take-make-dispose’ basis. Instead, it focuses on sustainable economic development by maximizing the reuse of materials and reducing environmental impacts [105].

**Fig. 2:**
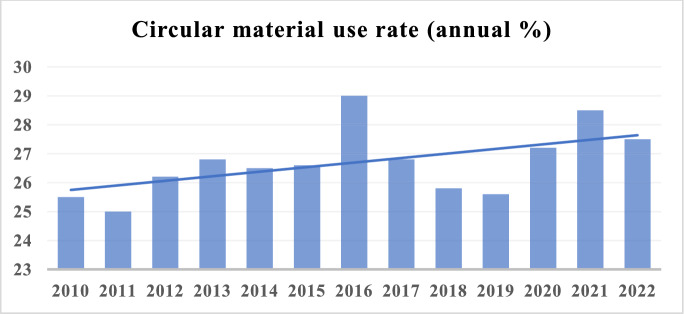
Circular material use rate (annual %) in the Netherlands. Source: Eurostat (https://ec.europa.eu/eurostat)

The Netherlands is among the leading nations in advancing circular economy principles, alongside the UK, Belgium, Spain, France, Italy, and Germany [70]. The Netherlands’ leadership in this area is well-established, having initiated circular economy activities as early as 2011. By 2013, the country had produced a pivotal report titled “Opportunities for a Circular Economy in the Netherlands” [12], marking a significant milestone in its commitment to this economic model. Figure 2 illustrates the circularity rate of the Dutch economy over the selected period, highlighting the progress made and the potential for further improvement.

The implementation of targeted strategies across four critical sectors—consumer goods, construction, polymers, manufacturing, and biomass and food—could potentially triple the national circularity rate. Such progress would be instrumental in helping the Netherlands achieve its goal of a fully circular economy by 2050 [48]. Moreover, the shift to a circular economy presents opportunities for stimulating economic development and creating new jobs through innovation and sustainable practices. Recognizing these benefits, the Netherlands launched a nationwide initiative in 2016, focusing on reducing the reliance on natural resource imports and enhancing sovereignty over raw material supplies. This approach not only contributes to environmental sustainability but also ensures greater security and resilience in the supply chain [86].

This study aims to strengthen our knowledge of the three main processes via which the circular economy affects GHG emissions in the Netherlands. First, circular economy techniques have a lot of promise in areas like transportation, the built environment, and food systems. They do this by reducing the need for new goods and virgin raw materials, which lowers emissions throughout manufacturing. Further potential to reduce emissions is provided by consumption-side policies and innovative product design, and operational energy savings may be realized in sectors like transportation and heating. Second, these tactics solve material management issues in mineral supply and trash from decommissioned technology while easing the shift to renewable energy sources like solar, wind, and electric cars. Third, while less studied, circular economy techniques may also help with climate adaptation by increasing flood resistance, promoting soil health, and delaying environmental deterioration. Although mitigation has been the main emphasis, a more thorough approach is required to properly grasp the potential of circular economy methods in climate adaptation. The study makes several key contributions to the existing literature. First, it focuses on the Netherlands, a leading European nation in the circular economy, and fills a gap in the research by using Dutch data to examine the impact of the circular economy on GHG emissions. This study investigates the relationship between GHG emissions and variables such as the circular economy, technological innovation, environmental taxes, economic instability, and industrialization in the Netherlands. Its primary objective is to provide insights into the policy implications for emission reduction initiatives in the Netherlands, particularly by exploring the interrelationships among these variables and their influence on GHG emissions. Second, the research employs the autoregressive distributed lag (ARDL) bounds test to examine the cointegration relationship between GHG emissions and specific variables, focusing on the long-run equilibrium. Additionally, the study uses Wavelet Coherence Analysis (WCA) to investigate the time–frequency dependence between GHG emissions and multiple factors, offering a novel approach in this context. As far as we know, no previous research has utilized WCA to explore the relationship and causality between GHG emissions and factors such as the circular economy, technological innovation, environmental taxes, economic instability, and industrialization. WCA provides a continuous representation of time series in the time–frequency domain, enabling a more profound understanding of these relationships.

The structure of this paper is as follows: "Literature review" section provides a comprehensive review of the literature; "Research methodology" section discusses the research methodology; "Results and discussions" section presents the main results and discusses their implications; and "Conclusions and policy implications" section concludes the research, offering recommendations for future studies and policy considerations.

## Literature review

To provide a comprehensive understanding of the complex interplay between economic activities, environmental policies, and technological advancements in the context of climate change, this literature review delves into the multifaceted impact of human activities on the environment.

### Human activities and environmental impacts

Human activities are intricately linked to both the exacerbation and mitigation of climate change, creating a complex and interconnected dynamic that shapes environmental outcomes. On one hand, the industrial and residential actions that drive economic growth and societal development have led to significant environmental degradation [74]. This includes pollution across various forms—air, CO_2_, water, and light—coupled with the heightened consumption of fossil fuels and the generation of GHG emissions. These processes not only contribute to global warming but also result in the accumulation of residential and industrial waste, which presents ongoing challenges in terms of effective waste management and environmental sustainability [83].

This narrative is not entirely one of environmental decline. Human activities have also sparked positive environmental changes, particularly through initiatives like recycling and waste sorting [59]. Recycling, for example, has emerged as a critical response to the environmental pressures brought about by mass manufacturing and the widespread use of materials like plastics throughout the twentieth century. Sustainable development goals can be achieved through innovation by fostering the development and implementation of eco-friendly technologies and practices that reduce environmental impact while promoting economic growth [15]. By prioritizing innovations in clean energy, resource efficiency, and sustainable production, green innovation aligns economic progress with environmental preservation, helping to meet global sustainability targets [21] and COP27 targets [94]. The benefits of recycling extend beyond merely reducing waste; it conserves valuable resources, curtails environmental degradation, and fosters a more sustainable interaction between human activities and the natural world [46].

In exploring the broader implications of these practices, Stahel [90] offers a profound analysis of circularity, positioning it as a fundamental strategy for achieving long-term economic and environmental sustainability. The concept of circularity—where resources are reused and repurposed to minimize waste—has garnered significant academic interest as a viable approach to restore both economic and ecological balance [41]. However, the bulk of the existing literature has predominantly concentrated on theoretical explorations of circularity, often neglecting empirical, data-driven analyses. This gap is largely due to the scarcity of comprehensive data at both the industrial and household levels, which limits the ability to fully assess the practical implications of circularity in real-world contexts.

Nonetheless, some empirical studies have made important strides in this area. For instance, Slorach et al. [89] conducted a thorough investigation into food waste treatment in the UK, demonstrating how circular economy principles can drive both environmental and economic sustainability. Their research highlights that employing anaerobic digestion for food waste not only mitigates environmental pollution but also enhances the quality of life and offers substantial economic advantages. Similarly, Gallego-Schmid et al. [38] examined the role of circular economy practices within the European Union’s construction industry—a sector known for its significant contribution to atmospheric CO_2_ and GHG emissions. Their findings underscore the crucial role of using recycled materials in improving resource efficiency and reducing environmental disparities across different EU member states.

### Circular economy and GHG emissions

Recycling and waste management practices have emerged as pivotal strategies in the global effort to combat climate change, offering significant environmental benefits that extend beyond mere waste reduction. Cudjoe et al. [24] underscore the crucial role that recycling plays in this context, highlighting its ability to substantially mitigate CO_2_ and methane emissions through the effective recycling of materials like steel, nonferrous metals, plastic, and paper. Their research illustrates the tangible impact that such practices can have on reducing GHG emissions, positioning recycling as a cornerstone of sustainable environmental management.

However, the narrative surrounding circular economy practices is not without its complexities. Li et al. [60] offer a nuanced perspective, examining the trade dynamics between China and Nigeria and the potential of the circular economy to reduce energy intensity in countries with prominent mining and extractive industries. Despite improvements in energy efficiency, their findings reveal a critical disconnect: the anticipated reduction in CO_2_ emissions did not materialize, underscoring the challenges and limitations of circular economy strategies in sectors with high energy demands. Similarly, energy security is required for countries (such as France, Germany, Japan, the United States, and the UK) to continue their sustainable energy policies [18]. Combustible renewables, waste, and modern renewables significantly contribute to environmental sustainability and the reduction of ecological footprints [78]. Their contribution highlights the need for a more tailored approach to implementing circular economy practices, one that accounts for the specific characteristics and constraints of different industries.

Adding another layer to this discussion, Bayar et al. [14] investigated the effects of renewable energy adoption and recycling waste management on environmental sustainability across EU member states. Their analysis, spanning from 2004 to 2017, yielded surprising results: no direct causality was found between recycling rates, renewable energy usage, and reductions in CO_2_ emissions. Similarly, Caglar et al. [20] indicate that solid waste management has a positive relationship with environmental quality and helps to achieve SDG-11 goals. This finding challenges the conventional wisdom that these practices are straightforward solutions to environmental challenges, suggesting instead that a more complex array of factors—including technological innovation, policy frameworks, and economic conditions—may play a more critical role in driving substantial emissions reductions.

Liu et al. [65] examine the development of China’s plastic recycling sector, emphasizing the intricate interactions between commercial, regulatory, and technical elements that affect the decrease of GHG emissions. The necessity for more accurate measurement of emission reductions via circular economy methods is also emphasized by Gallego-Schmid et al. [38], who call for a sophisticated methodology that considers the circumstances and trade-offs involved. The transition of Denmark’s waste management industry from a linear to a circular economy is examined by Magazzino et al. [67], who highlight the policy implications for lowering emissions. The potential of circular economy techniques to mitigate climate change internationally is further supported by research from 29 European nations presented by Hailemariam and Erdiaw-Kwasie [47], which demonstrates a substantial association between the advancement of the circular economy and lower CO_2_ emissions.

### Technological innovation, environmental tax, economic instability, and industrialization

Technological innovation is another critical variable that influences the relationship between the circular economy and GHG emissions. Technological advancements have the potential to either increase or decrease global GHG emissions, depending on how they are implemented. Following the Environmental Kuznets Curve (EKC) theory, emissions first increase as economies expand and industrialize, but once a certain degree of economic development and innovation is attained, emissions eventually fall as cleaner technologies and more effective production techniques are adopted. Houston and Reay [50] noted that while technological innovation is a growing source of emissions from energy and manufacturing sectors throughout its lifecycle phases, it also offers the potential to significantly reduce emissions in sectors like transportation by circumventing traditional energy-intensive processes. In order to accelerate the adoption of sustainable technologies that eventually reduce emissions, the ‘Diffusion of Innovations Theory’ highlights the need for policy, market incentives, and social acceptability. It also explains how innovative technologies spread throughout sectors [39]. Using the Wavelet Quantile-on-Quantile Regression (WQQR) approach, Ozkan et al. [76] indicated that green technologies mitigate CO_2_ emissions in Germany for the period of 1974 to 2019. Malmodin et al. [69] further suggested that technological innovation can have both positive and negative effects on GHG emissions. Appiah-Otoo et al. [9] demonstrated that technological innovation enhances environmental sustainability in countries with high innovation quality but can have detrimental effects in nations with lower innovation quality. Their causality study revealed a two-way causal relationship between technological innovation and CO_2_ emissions in countries with high and moderate innovation quality, but only a one-way causal relationship from CO_2_ emissions to technological innovation in countries with poor innovation quality. Amari et al. [8] found that greater technological readiness, use, and intensity can reduce CO_2_ emissions and energy consumption, thereby improving environmental sustainability in lower- and lower-middle-income countries.

Environmental taxes are another important factor in the circular economy-GHG emissions nexus. In this context, the ‘Pigovian Taxation’ is foundational, positing that environmental taxes, such as carbon taxes, internalize the external costs of pollution by making carbon-intensive activities more expensive, thus encouraging businesses and consumers to reduce emissions and adopt cleaner alternatives [28]. The effectiveness of environmental taxes, including CO_2_ and energy taxes, in reducing GHG emissions has been widely recognized. Ghazouani et al. [42] highlighted the effectiveness of environmental taxes and the promotion of cleaner energy sources in reducing pollution in nine leading European countries. Similarly, Hsu et al. [51] emphasized the crucial role of environmental taxes in mitigating GHG emissions in China. Their study revealed that environmental taxes could significantly reduce haze pollution, particularly PM2.5 levels, in China. Doğan et al. [26] discussed the potential benefits of environmental taxes in reducing the impact of energy consumption and natural resource usage while promoting renewable energy sources. These measures positively affect the environment and help mitigate GHG emissions. Wolde-Rufael and Mulat-Weldemeskel [98] also found a significantly negative association between environmental taxes and CO_2_ emissions across 20 European countries. Moreover, to provide a double dividend of economic and environmental advantages, ‘Environmental Tax Reform’ theory goes one step further and proposes that the money collected from environmental taxes be reinvested in renewable energy, green technology, or tax breaks elsewhere, as suggested by Ekins et al. [29].

Economic instability is another variable considered in the relationship between the circular economy and GHG emissions. The study used bank non-performing loans (NPLs) as a proxy to measure economic instability. Economic instability can negatively impact a nation’s overall economic development, leading to business difficulties, reduced investments, and fewer employment opportunities [98]. Economic stability is necessary to allocate funds to mitigate GHG emissions, as Emre Caglar et al. [30] argue that improving environmental quality requires countries to prioritize development investments, particularly by increasing their research and development budgets. This investment is crucial for advancing the transition to a low-carbon economy through innovation in sustainable technologies and practices. Moreover, as suggested by Caglar et al. [17], economic well-being enhances income quality and eventually leads to improved environmental outcomes. As income rises, the capacity for investing in cleaner technologies and sustainable practices increases, promoting environmental quality. This also supports the EKC hypothesis.

Finally, industrialization is a key variable in examining the link between the circular economy and GHG emissions. Industrialization has been largely acknowledged as a major catalyst of climate change due to the significant CO_2_ emissions and other GHG released into the atmosphere as a result of human activity during the Industrial Revolution. Aslam et al. [10] illustrated the positive and bidirectional causal relationship between industrialization and CO_2_ emissions in China. Azam et al. [11] found that industrialization leads to an increase in environmental pollution through GHG emissions. Moreover, energy production, transportation, industry, agriculture, and construction are some of the key industries that contribute significantly to emissions as a result of industrialization. Due to its reliance on fossil fuels, the energy industry is a significant emitter, and transportation also contributes through the use of fuel in cars and airplanes [33].

## Research methodology

### Theoretical framework

The linear economy, characterized by its "take-make-dispose" model, relies on the extraction of raw materials, production, consumption, and disposal of products, leading to the depletion of natural resources and environmental degradation [71]. This traditional model focuses on financial gains and consumer convenience, often disregarding the long-term ecological consequences, with products designed for single use and waste generation. In contrast, the circular economy offers a paradigm shift by promoting resource efficiency and minimizing waste through practices like recycling, reusing, and remanufacturing. Transitioning to a circular economy, however, requires overcoming challenges such as technological limitations, economic barriers, and shifts in consumer behavior [82], all of which must be addressed to ensure successful implementation.

**Fig. 3:**
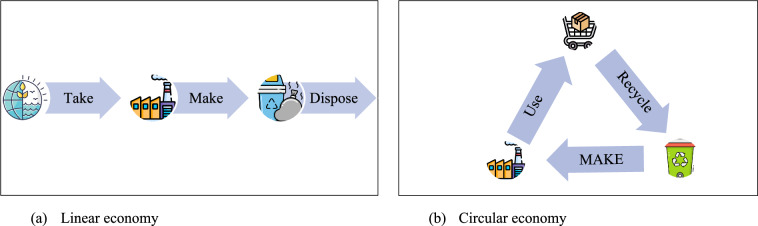
Linear economy vs circular economy. **a** Linear economy, **b** circular economy

The circular economy emphasizes sustainability by optimizing resource use, reducing waste, and minimizing emissions from resource extraction and processing [63]. This model involves innovative approaches to maintain environmental balance and addresses not only ecological concerns but also social factors like human well-being [31]. Implementing a circular economy requires a collaborative effort among governments, corporations, and consumers to drive regulatory frameworks, innovative business practices, and sustainable consumption behaviors [72, 77]. Ultimately, the transition to a circular economy offers a more sustainable alternative to the linear model [74], fostering long-term ecological balance while reducing environmental impacts (see Fig. 3 for a comparison of linear and circular economies).

### Model specifications

In alignment with the methodologies employed by Shahzad et al. [85] and Wang et al. [96], our study utilizes a modified model to investigate the impact of circular economy practices on GHG emissions. While previous studies predominantly focused on CO_2_ emissions as the dependent variable, our analysis expands this scope by using GHG emissions to capture a more comprehensive range of emissions affecting climate change. Additionally, whereas many studies have used waste management metrics to represent circular economy practices, our research adopts the circular material use rate as the primary independent variable. This adjustment allows us to directly evaluate how the extent of material circularity influences overall GHG emissions. The basic model is expressed as Eq. 1.

1 $${\text{GHG emissions}}_{\text{t}} = \vartheta_{0} + \vartheta_{1} {\text{GHG emissions}}_{{\text{t}} - 1} + \vartheta_{2} {\text{Circular economy}}_{{\text{t}}} + \vartheta_{3} {\text{Technological innovation}}_{{\text{t}}} + \vartheta_{4} {\text{Environmental tax}}_{{\text{t}}} + \vartheta_{5} {\text{Economic instability}}_{{\text{t}}} + \vartheta_{6} {\text{Industralization}}_{{\text{t}}} + \varepsilon_{{\text{t}}}$$

where $\vartheta_{0}$ denotes the constant term, whereas $\vartheta_{1} , \cdots ,\vartheta_{6}$ represent the parameters that need estimation.

Furthermore, whereas many studies have utilized waste management as an indicator of circular economy practices [89], this study adopts the circular material use rate as the independent variable to more accurately reflect material circularity. The choice of this variable is informed by Wolde-Rufael and Mulat-Weldemeskel [98], who argue that circular material use rate provides a clearer picture of resource efficiency and recycling efforts.

The dataset employed consists of quarterly observations from the Netherlands, covering the period from 2010Q1 to 2022Q2. To convert annual data into a quarterly frequency, we applied the quadratic match-sum method, as described by Shahbaz et al. [84]. This method ensures a detailed temporal resolution of the data, aligning with practices outlined by Dehghan Shabani and Shahnazi [25]. All variables were transformed into their natural logarithmic form, enhancing the accuracy of the analysis and allowing for clear interpretation of the coefficients as elasticities, representing proportional differences in the context of the study [44].

The dependent variable, GHG emissions, is measured in tons per capita and sourced from the Eurostat database. This metric provides a comprehensive view of the total emissions produced by a country, echoing the findings of Magazzino et al. [67], who highlighted the importance of using comprehensive emission indicators.

This study assesses the impact of circular economy practices by focusing on the circular material use rate, a data source from Eurostat. This rate shows how much recycled materials are put back into the economy as a percentage of all the materials that are used. It shows how well efforts to promote circularity are working to lower our need for new raw materials [38].

In addition to focusing on the primary variables, this study integrates several control factors to thoroughly examine their potential impact on GHG emissions. The role of technological innovation is captured through the gross value added in the information and communication technologies (ICT) sector, which includes both manufacturing and services. This measure reflects the complex effects of technological advancements: while innovations in manufacturing may contribute to increased emissions due to higher energy consumption [79], advancements in services have the potential to enhance efficiency and reduce resource use, as noted by Abbas, et al. [1]. Similarly, Caglar et al. [16] demonstrate that environmental quality increases when industries are competitive and technological advancement is high.

We incorporate the influence of environmental tax revenue to evaluate its effect on GHG emissions. Measured as a percentage of total tax and social contribution revenue, this tax aims to incentivize emission reductions by imposing a fee on each ton of GHG and CO_2_ emitted. The goal is to encourage more cost-effective abatement measures, thereby aligning with the findings of Ghazouani et al. [43], who emphasize the role of taxation in driving environmental improvements.

Bank non-performing loans, expressed as a percentage of total loans, serve as a proxy for economic instability. This variable provides insight into how financial stability fluctuations can impact investments in sustainable practices [103, 104]. The connection between bank loans and emissions illustrates the evolving role of financial institutions in promoting environmentally friendly investments, a trend highlighted by Abbas et al. [2]. This variable helps understand how financial disruptions can influence the capacity for green investments and sustainability efforts.

Finally, industrialization is evaluated through the value added in the industrial sector, including construction, as a percentage of GDP. The relationship between industrialization and GHG emissions is multifaceted: early stages of industrial development are often associated with increased emissions due to greater fossil fuel use [102].

These considerations are summarized and detailed in Table 1, providing a comprehensive view of the variables and their interconnections within the model.

**Table 1:** Variables description

Variable	Description	Units	Source
GHG emissions	Net greenhouse gas emissions	Tones per capita	Eurostat
Circular economy	Circular material use rate	Annual %	Eurostat
Technology innovation	Technological innovation service (gross value added)	% of the technological innovation sector in gross value added	Eurostat
Environmental tax	Environmental tax revenue	% of total revenue from taxes and social contributions	Eurostat
Economic instability	Bank non-performing loans	% of total gross loans	WDI
Industrialization	Industry value added	% of GDP	WDI

### Methodological framework

The methodological framework outlines the approach used to analyze the relationship between GHG emissions and the various factors discussed previously. By employing sophisticated econometric techniques, the study aims to ensure robust and reliable results. This framework integrates both primary and control variables, facilitating a comprehensive evaluation of their impacts on environmental outcomes.

**Fig. 4:**
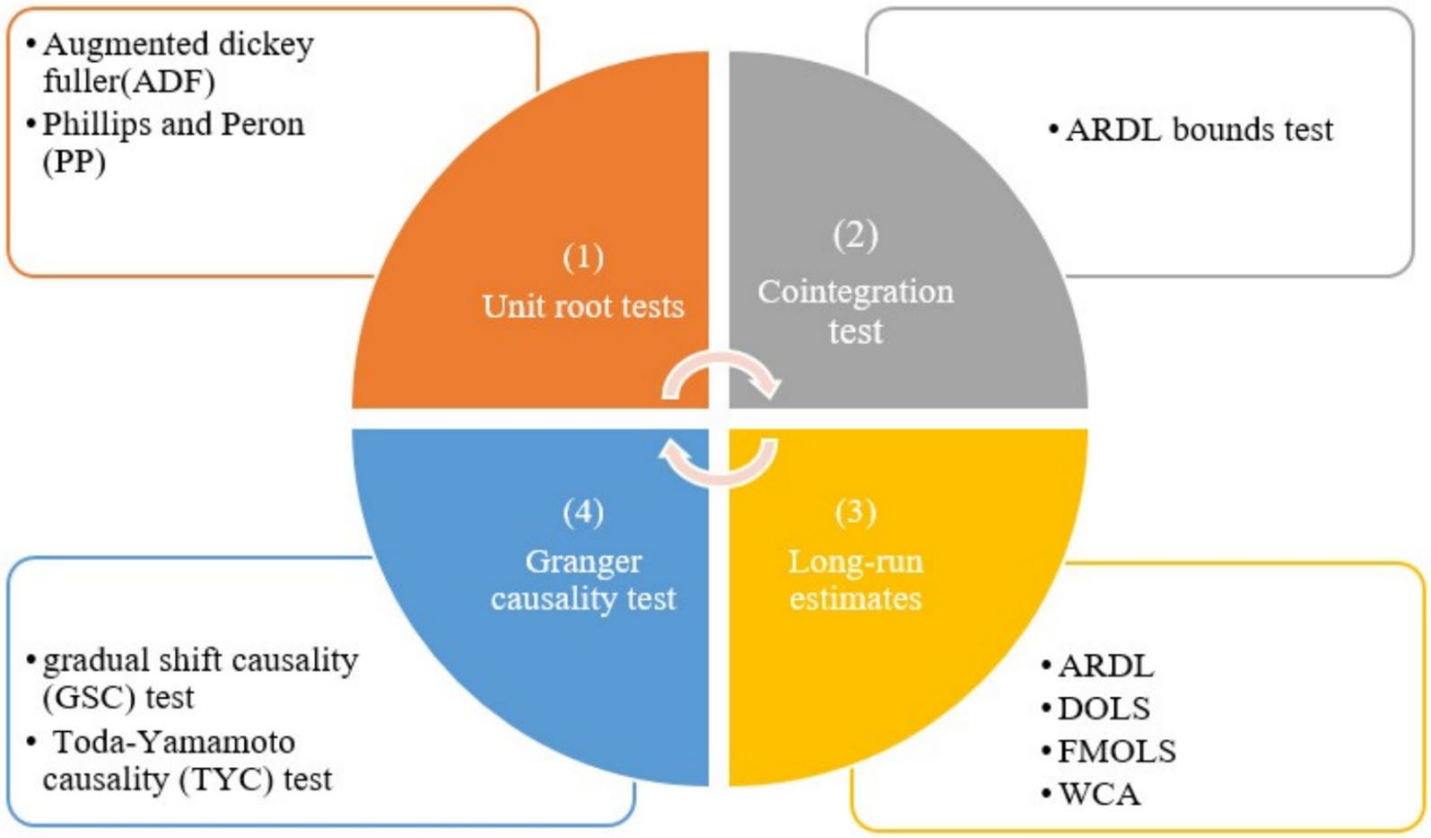
Methodological framework

This section delineates the econometric methods employed in this study, as illustrated in Fig. 4.

Unit root tests are fundamental in time series analysis, providing critical insights into the stationarity properties of the data. Stationarity is a key assumption in time series modeling, influencing the accuracy of statistical inferences and forecasts. The primary tests used for detecting unit roots are the Augmented Dickey-Fuller (ADF) test and the Phillips-Perron (PP) test. These tests help researchers understand the temporal behavior of a series and guide the selection of appropriate models for analysis. By identifying unit roots, these tests prevent misleading regression results and ensure that statistical analyses are based on sound assumptions.

The ADF test is defined by the following Eq. 2:

2 $$\Delta {\text{Y}}_{{\text{t}}} = \beta {\text{D}}_{{\text{t}}} + \tau {\text{Y}}_{{\text{t}} - 1} + \mathop \sum \limits_{{\text{j}} - 1}^{{\text{p}}} \sigma_{{\text{j}}} \Delta {\text{Y}}_{{\text{t}} - {\text{j}}} + \varepsilon_{{\text{t}}}$$

Where D_t_ represents the deterministic term vector, and ${\upvarepsilon }_{\text{t}}$ is the error term. Similarly, the PP test is expressed as in Eq. 3:

3 $$\Delta {\text{Y}}_{{\text{t}}} = \beta {\text{D}}_{{\text{t}}} + \tau {\text{Y}}_{{\text{t}} - 1} + \varepsilon_{{\text{t}}}$$

Pesaran et al. [80] developed the ARDL bounds testing approach, which offers several advantages over traditional cointegration tests, primarily its flexibility in handling series integrated with different orders, whether I(0) or I(1). The ARDL method allows for the derivation of the unrestricted error correction model (ECM), which captures both short-term and long-term dynamics within the data. This advantage makes it particularly useful even with relatively small sample sizes, producing consistent and reliable results [32, 34, 35]. To determine the maximum lag length for each variable, the ARDL approach uses (P + 1)^k^ regressions, where p is the maximum number of lags and k is the number of variables in the equation. The lag selection criteria are based on the Akaike Information Criterion (AIC) and Schwarz Bayesian Criterion (SBC). The joint F-statistic evaluates the null hypothesis of no cointegration [7].

In the context of this study, the ARDL bounds testing is applied to evaluate the cointegration relationship among GHG emissions and various independent variables. The methodology begins with the inclusion of lagged levels of the independent variables in the ARDL equation and subjecting them to an F-test to determine the presence of a long-run relationship [33]. The empirical application of this approach is illustrated by the following Eq. 4:

4 $$\Delta {\text{lnGHG emissions}}_{{\text{t}}} = \vartheta_{0} + \mathop \sum \limits_{{{\text{I}} = 1}}^{{\text{n}}} \vartheta_{{1{\text{i}}}} \Delta {\text{lnGHG emissions}}_{{{\text{t}} - {\text{i}}}} + \mathop \sum \limits_{{{\text{I}} = 0}}^{{\text{n}}} \vartheta_{{2{\text{i}}}} \Delta {\text{lnCircular economy}}_{{{\text{t}} - {\text{i}}}} + \mathop \sum \limits_{{{\text{I}} = 0}}^{{\text{n}}} \vartheta_{{3{\text{i}}}} \Delta {\text{lnTechnological innovation}}_{{{\text{t}} - {\text{i}}}} + \mathop \sum \limits_{{{\text{I}} = 0}}^{{\text{n}}} \vartheta_{{4{\text{i}}}} \Delta {\text{lnEnvironmental tax}}_{{{\text{t}} - {\text{i}}}} + \mathop \sum \limits_{{{\text{I}} = 0}}^{{\text{n}}} \vartheta_{{5{\text{i}}}} \Delta {\text{lnEconomic instability}}_{{{\text{t}} - {\text{i}}}} + \mathop \sum \limits_{{{\text{I}} = 0}}^{{\text{n}}} \vartheta_{{6{\text{i}}}} \Delta {\text{lnIndustralization}}_{{{\text{t}} - {\text{i}}}} + \xi_{1} {\text{lnGHG emissions}}_{{{\text{t}} - 1}} + \xi_{2} {\text{lnCircular economy}}_{{{\text{t}} - 1}} + \xi_{3} {\text{lnTechnological innovation}}_{{{\text{t}} - 1}} + \xi_{4} {\text{lnEnvironmental tax}}_{{{\text{t}} - 1}} + \xi_{5} {\text{lnEconomic instability}}_{{{\text{t}} - 1}} + \xi_{6} {\text{lnIndustralization}}_{{{\text{t}} - 1}} + \varepsilon_{t}$$

In this equation, $\vartheta_{0}$ represents the intercept, while ξ_1_ to ξ_6_, and $\vartheta_{1}$ to $\vartheta_{6}$ are the long- and short-run coefficients, respectively.

The null and alternative hypotheses for cointegration in this context are:

$$\begin{gathered} {\text{H}}_{0} : \xi_{1} = \xi_{2} = \xi_{3} = \xi_{4} = \xi_{5} = \xi_{6} = 0 \hfill \\ {\text{H}}_{{\text{a}}} : \xi_{1} \ne \xi_{2} \ne \xi_{3} \ne \xi_{4} \ne \xi_{5} \ne \xi_{6} \ne 0 \hfill \\ \end{gathered}$$

Choosing the correct lag length is crucial for accurately capturing variable dynamics. An incorrect lag length can result in biased estimates and false conclusions about long-run relationships. Careful selection ensures reliable findings and avoids misspecification. The error correction model estimates short-run relationships as given by Eq. 5:

5 $$\Delta {\text{lnGHG emissions}}_{{\text{t}}} = \vartheta_{0} + \mathop \sum \limits_{{{\text{I}} = 1}}^{{\text{n}}} \vartheta_{{1{\text{i}}}} \Delta {\text{lnGHG emissions}}_{{{\text{t}} - {\text{i}}}} + \mathop \sum \limits_{{{\text{I}} = 0}}^{{\text{n}}} \vartheta_{{2{\text{i}}}} \Delta {\text{lnCircular economy}}_{{{\text{t}} - {\text{i}}}} + \mathop \sum \limits_{{{\text{I}} = 0}}^{{\text{n}}} \vartheta_{{3{\text{i}}}} \Delta {\text{lnTechnological innovation}}_{{{\text{t}} - {\text{i}}}} + \mathop \sum \limits_{{{\text{I}} = 0}}^{{\text{n}}} \vartheta_{{4{\text{i}}}} \Delta {\text{lnEnvironmental tax}}_{{{\text{t}} - {\text{i}}}} + \mathop \sum \limits_{{{\text{I}} = 0}}^{{\text{n}}} \vartheta_{{5{\text{i}}}} \Delta {\text{lnEconomic instability}}_{{{\text{t}} - {\text{i}}}} + \mathop \sum \limits_{{{\text{I}} = 0}}^{{\text{n}}} \vartheta_{{6{\text{i}}}} \Delta {\text{lnIndustralization}}_{{{\text{t}} - {\text{i}}}} + \varrho {\text{ECM}}_{{{\text{t}} - {\text{i}}}} + \varepsilon_{{\text{t}}}$$

### Robust analyses

For robustness, we employed the fully modified ordinary least squares (FMOLS), dynamic ordinary least squares (DOLS), and wavelet coherence analysis to check the consistency of ARDL bounds testing.

#### FMOLS and OLS

FMOLS and DOLS represent sophisticated time-series techniques employed for cointegration analysis, effectively tackling challenges such as serial correlation and endogeneity. FMOLS addresses the issues of serial correlation and endogenous regressors by making adjustments to the estimator’s bias, whereas DOLS utilizes leads and lags of the first-differenced regressor to mitigate potential endogeneity and serial correlation. These methods outperform traditional OLS as they yield more consistent and efficient estimates when addressing non-stationary, cointegrated time series, thus enhancing the reliability of long-term relationship analysis.

6 $$\hat{\beta }^{*}_{{\text{FMOLS or DOLS}}} = {\text{N}}^{ - 1} \mathop \sum \limits_{{{\text{i}} = 1}}^{{\text{N}}} \hat{\beta }^{*}_{{\text{FMOLS or DOLS}}}$$

where $\hat{\beta }^{*}_{{\text{FMOLS or DOLS}}}$ are estimators FMOLS and DOLS conventionally. Moreover, the t-statistic associated the estimators can be obtained as given by Eq. 7:

7 $${\text{t}}_{{\hat{\beta }^{*}_{{\text{FMOLS, DOLS}}} }} = {\text{N}}^{{ - \frac{1}{2}}} \mathop \sum \limits_{{{\text{i}} = 1}}^{{\text{N}}} {\text{t}}_{{\hat{\beta }^{*}_{{\text{FMOLS, DOLS}}} }}$$

#### Wavelet coherence analysis

Wavelet coherence analysis has become increasingly significant in economics, providing a sophisticated tool for examining the relationships, interactions, and dynamics between economic time series data. Originating from the work of Goupillaud et al. [45], this method has been pioneering in its application across various fields, including economics. Given that economic variables often exhibit time-varying relationships, wavelet coherence allows researchers to analyze how coherence and phase relationships between different economic indicators evolve over time and across different frequency components. Wavelet coherence analysis not only provides access to the local phase connection of time sequences in the time–frequency field but also identifies noteworthy correlations [99]. Wavelet coherence analysis allows for the extraction of correlation characteristics throughout a wider frequency range, unlike cross-wavelet analysis, which is limited to certain frequency points due to scale smoothing [37]. This capability is crucial for understanding the cyclical patterns and interactions within economic data, as highlighted by Abbas et al. [3]. The application of wavelet coherence analysis begins with the use of the Morlet wavelet family, represented mathematically as:

8 $$\varpi ({\text{t}})={\pi }^{-\frac{1}{4}}{{\text{e}}}^{- {\text{i}} \varpi {\text{t}}}{{\text{e}}}^{-\frac{1}{2}{{\text{t}}}^{2}}$$

By transforming the wavelet ψ(t), the expression for ψk, f(t) is derived, as shown in:

9 $${\varpi }_{{\text{k,f}}} ({\text{t}})=\frac{1}{\sqrt{{\text{h}}}}\varpi \left(\frac{{\text{t}}-{\text{k}}}{{\text{f}}}\right), \;\;\; {\text{k,f}}\in {\mathbb{R}},{\text{f}}\ne 0$$

The cross wavelet transform (CWT) is a fundamental component of this analysis and is given by:

10 $${\varpi }_{{\text{p}}}\left({\text{k,f}}\right)={\int }_{-\infty }^{\infty }{\text{p}}({\text{t}})\frac{1}{\sqrt{{\text{f}}}}\varpi \left(\frac{{\overline{{\text{t}}-{\text{k}}}}}{{\text{f}}}\right){\text{dt}},\; {\text{N}}$$

Further refinement of the process involves adding a coefficient ψ to obtain p(p(t), which is represented by:

11 $${\text{p}}({\text{t}})=\frac{1}{{{\text{C}}}_{\varpi }}{\int }_{0}^{\infty }\left[{\int }_{-\infty }^{\infty }{\left|{{\text{W}}}_{{\text{p}}}({\text{a,b}})\right|}^{2}{\text{da}}\right]\frac{{\text{db}}}{{{\text{b}}}^{2}}$$

The wavelet power spectrum (WPS) then illustrates the vulnerability of the time series, capturing its dynamics as shown in:

12 $${{\text{WPS}}}_{{\text{p}}}({\text{k,f}})={\left|{{\text{W}}}_{{\text{p}}}({\text{a,b}})\right|}^{2}$$

The cross wavelet transform of time series, specifically for variables like GHG emissions, circular economy, technological innovation, environmental tax, economic instability, and industrialization in the Netherlands over the period from 2010Q1 to 2022Q4, is denoted by:

13 $${{\text{W}}}_{{\text{pq}}}({\text{k,f}})={{\text{W}}}_{{\text{p}}} ({\text{k,f}})\overline{{{\text{W}}}_{{\text{q}}}({\text{k,f}})}$$

The interaction between these variables is then quantified through the squared wavelet coherence R^2^(k,f), defined as:

14 $${{\text{R}}}^{2}({\text{k,f}})=\frac{{\left|{\text{C}}\left({{\text{f}}}^{-1}{{\text{W}}}_{{\text{pq}}} ({\text{k,f}})\right)\right|}^{2}}{{{\text{C}}({\text{f}}}^{-1}{\left|{{\text{W}}}_{{\text{p}}} ({\text{k,f}})\right|}^{2}) {{\text{C}} ({\text{f}}}^{-1}{\left|{{\text{W}}}_{{\text{q}}} ({\text{k,f}})\right|}^{2})}$$

Here, the R^2^(k,f) value serves as an indicator of the strength and magnitude of the interaction between the economic time series. Adebayo and Odugbesan [4] further explored the coherence between variables using lags in two-time series, offering a linked orientation argument that adds depth to the analysis. The phase difference between the series is captured through the phase angle, denoted by:

15 $${\varphi }_{{\text{pq}}}({\text{k,f}})={\tan}^{-1}\left(\frac{{\text{L}}\left\{{\text{C}}\left({{\text{f}}}^{-1}{{\text{W}}}_{{\text{pj}}} ({\text{k,f}})\right)\right\}}{{\text{O}}\left\{{\text{C}}\left({{\text{f}}}^{-1}{{\text{W}}}_{{\text{pj}}} ({\text{k,f}})\right)\right\}}\right)$$

This robust test, employed as a supplementary method alongside the ARDL approach, provides a comprehensive framework for analyzing the intricate relationships between economic indicators over time and across varying frequencies, thus enhancing the robustness and reliability of the econometric analysis.

#### Causality analysis

The Toda and Yamamoto [92] methodology enhances traditional Granger causality testing by allowing for causality assessment between variables, regardless of their integration order. This method is crucial in addressing the problem of spurious causality, which can arise from non-stationary data or omitted variables. The Toda-Yamamoto method makes sure that causality results are more solid and reliable by using an augmented vector autoregression (VAR) model with extra lags. This method also effectively handles endogeneity, capturing the bidirectional feedback effects often observed in economic variables and providing a more comprehensive understanding of their interactions. Moreover, the Gradual Shift Causality test and the Toda-Yamamoto test are advanced methods for analyzing causal relationships in time series data that address limitations of the traditional Granger causality test. The Gradual Shift Causality test is designed to handle situations where the relationship between variables may change over time, accounting for non-linear dynamics and structural breaks that could be overlooked by traditional methods. On the other hand, the Toda-Yamamoto test improves upon the Granger causality test by allowing for variables that are integrated in different orders (such as I(0) or I(1)) without the need for pre-testing or differencing, making it more robust in the presence of unit roots or non-stationarity. Both tests offer greater flexibility and robustness, providing more reliable results in complex data structures where traditional Granger causality may fail to capture the true causal relationships [32].

## Results and discussions

In this study, statistical measures like skewness and kurtosis were employed to assess the normality of the data, providing insights into potential deviations from a normal distribution. To rigorously examine the normality of the variables, the Jarque and Bera [57] test was applied. The results, as presented in Table 2, indicate that all variables exhibit non-linear behavior, suggesting deviations from normality.

**Table 2:** Summary statistics

Variables	ln GHG emissions	lnCircular economy	lnEconomic instability	lnEnvironmental tax	lnTechnology innovation	lnIndustrialization
Mean	2.468	3.284	0.857	2.149	1.548	2.923
Median	2.502	3.279	0.933	2.168	1.544	2.904
Maximum	2.665	3.373	1.178	2.293	1.620	3.010
Minimum	2.207	3.215	0.450	1.690	1.490	2.865
Std. Dev	0.108	0.043	0.235	0.118	0.033	0.051
Skewness	−0.726	0.457	−0.242	−1.968	0.469	0.447
Kurtosis	2.686	2.406	1.576	7.543	2.553	1.579
Jarque–bera	4.783	2.575	4.903	78.285	2.338	6.106
Probability	0.091	0.276	0.086	0.000	0.311	0.047
Obs	52	52	52	52	52	52

The stationarity of variables was assessed using the Augmented Dickey-Fuller (ADF) and Phillips-Perron (PP) tests before conducting ARDL bounds testing to ensure the integration order was suitable. The results, as shown in Table 3, confirm that all variables— GHG emissions, circular economy, economic instability, environmental tax, technological innovation, and industrialization—are non-stationary at their levels I(0), but become stationary after taking their first differences I(1).

**Table 3:** Unit root test

Variables	ADF test		PP test	
	Level	First difference	Level	First difference
lnGHG emissions	−1.819	−3.936***	−1.936	−3.832***
lnCircular economy	−1.554	−3.568***	−1.709	−3.482***
lnEconomic instability	−1.782	−3.787***	−1.929	−4.192***
lnEnvironmental tax	−1.683	−3.698***	−1.710	−3.642***
lnTechnological innovation	−1.851	−4.002***	−1.795	−4.279***
lnIndustrialization	−1.823	−3.916***	−1.882	−4.073***

***p < 0.01

Before applying the ARDL technique to estimate the model, it is essential to determine the appropriate lag length for the variables. The Akaike Information Criterion (AIC) was used to identify the optimal lag length, ensuring accurate model specification. The results of the ARDL cointegration test, presented in Table 4, indicate a significant long-run relationship among GHG emissions, circular economy, technological innovation, environmental tax, economic instability, and industrialization in the Netherlands. The F-statistic of 7.002, which exceeds the critical values at the 1, 5, and 10% significance levels, confirms the existence of cointegration among the variables, suggesting that these factors are interconnected in the long term.

**Table 4:** Cointegration test

Model	Optimal lag length	F-statistics	Cointegration
lnGHG emissions = f(lnCircular economy, lnTechnological innovation, lnEnvironmental tax, lnEconomic instability, lnIndustrialization)	(1,1,0,1,1,1)	7.002***	Yes
Significance level	I (0)	I (1)	
1%	3.21	4.19	
5%	2.33	3.41	
10%	2.01	3.02	

Based on the Akaike Information Criterion and intercept only. The period of analysis is 2010Q1–2022Q4. Maximum lag = 2, n = 52. The critical values for the lower I(0) and upper I(1) bounds were taken from Narayan [73]. ***p < 0.01

After establishing the presence of a long-run equilibrium relationship between the circular economy and the associated factors, this research delves into the short-run and long-run dynamics between GHG emissions and variables such as the circular economy, technological innovation, environmental tax, economic instability, and industrialization [6, 23]. The findings reveal several key insights with significant implications for sustainability and policy-making.

The results indicate a negative long-run relationship between the circular economy and GHG emissions, such that a 1% increase in circular economy results in a decrease of 0.540% of GHG emissions. This suggests that as the circular economy develops, GHG emissions decrease, highlighting the effectiveness of circular economy practices in mitigating environmental impact. The results align with previous research, such as the work of Hailemariam and Erdiaw-Kwasie [47] and Gallego-Schmid et al. [38], which similarly found that circular economy initiatives play a critical role in reducing environmental footprints. Our study contradicts the findings of Eweade et al. [32], who showed that the use of combustible renewable waste adversely affects the ecological footprint in the UK. The Netherlands’ commitment to transitioning to a fully circular economy by 2050 reflects this understanding, with strategies focused on reducing material consumption, promoting sustainable alternatives, extending product lifecycles, and enhancing recycling processes. These efforts are not only crucial for lowering GHG emissions but also for creating a more sustainable economic framework that balances resource efficiency with environmental stewardship. Moreover, by altering consumer behavior, legislative incentives, and production methods, the circular economy lowers GHG emissions. The circular economy decreases waste and the need for resource extraction and energy-intensive production, which lowers emissions, by designing items for longevity, repair, and recyclability. Carbon taxes, extended producer responsibility, and recycling requirements are a few examples of policy incentives that push companies to implement emission-reducing, sustainable practices. Furthermore, consumers are choosing services over ownership or reusing, repairing, and recycling objects, which reduces the need for new products and the emissions associated with their manufacture [54]. When combined, these processes provide a more sustainable and effective system that reduces GHG emissions throughout the course of a product’s lifespan.

Technological innovation also has a negative long-run relationship with GHG emissions, such that a 1% increase in technological innovation results in a decrease of 0.607% of GHG emissions, indicating that advancements in technology are pivotal in reducing the country’s carbon footprint and achieving environmental sustainability. This finding supports the views of Adebayo et al. [5], who emphasize the role of technological innovation in enhancing efficiency, reducing energy consumption, and substituting traditional methods with more sustainable alternatives. Different types of innovations, such as clean energy technologies and energy efficiency improvements, help reduce GHG emissions in complementary ways. By substituting low- or zero-emission energy sources for fossil fuel-based ones, clean energy technologies such as solar, wind, and geothermal power directly lower emissions from the production of electricity. Conversely, energy efficiency enhancements like improved insulation, energy-efficient appliances, and smarter systems lower the total amount of energy required for industrial operations, heating, and cooling. This lowers the demand for electricity and, consequently, lowers emissions from power generation. By addressing emissions from both the supply (clean energy) and demand (efficiency) sides, these advances collectively offer a comprehensive strategy for reducing climate change. In the Netherlands, the adoption of digital technologies and innovative solutions is projected to significantly cut down on GHG emissions. By improving energy management, optimizing transportation systems, and advancing industrial processes, technological innovation can lead to considerable reductions in environmental impact, underscoring its importance in the nation’s sustainability agenda.

Furthermore, the study finds that there is a negative long-run relationship between environmental taxes and GHG emissions, such that a 1% increase in environmental taxes results in a decrease of 0.531% of GHG emissions. The results indicate that the implementation of environmental taxes is associated with a reduction in GHG emissions. This finding is consistent with the studies such as Doğan et al. [26] and Ghazouani et al. [42], which demonstrate the efficacy of environmental taxes in promoting cleaner energy use and reducing reliance on fossil fuels. The gradual tightening of these tax policies, as seen in the Tax Plan 2023, further illustrates the Dutch commitment to achieving long-term environmental goals [55]. This suggests that such fiscal measures effectively incentivize businesses and individuals to reduce their carbon emissions by adopting cleaner practices and investing in energy efficiency. The Dutch government’s introduction of a GHG tax as part of its environmental protection strategy illustrates this principle in action. By taxing high-emission industries and encouraging the adoption of carbon capture and storage technologies, the Netherlands is curbing emissions and fostering a more sustainable industrial sector (Table 5).

**Table 5:** Long-run and short-run results

Variable	Coefficient	t-statistics	Prob
Dependent variable: lnGHG emissions			
Long-run estimation			
lnCircular economy	−0.540***	−5.703	0.000
lnTechnological innovation	−0.607***	−5.370	0.000
lnEnvironmental tax	−0.531***	−5.175	0.000
lnEconomic instability	0.099***	2.842	0.007
lnIndustrialization	0.517***	3.107	0.003
Short-run estimation			
lnCircular economy	−0.253***	−6.929	0.000
lnTechnological innovation	−0.363***	−6.687	0.000
lnEnvironmental tax	−0.077**	−2.382	0.022
lnEconomic instability	0.093***	2.833	0.008
lnIndustrialization	0.083**	2.632	0.019
Constant	−0.582***	−3.365	0.001
ECM(−1)	−0.842***	−5.558	0.000
R^2^	0.998		
Adjusted R^2^	0.998		
χ^2^ ARCH	2.127 (0.126)		
χ^2^ RESET	1.735 (0.139)		
χ^2^ normality	0.442 (0.531)		
χ^2^ LM	0.185 (0.728)		

χ^2^ ARCH for autoregressive conditional heteroscedasticity, χ^2^ Reset for Ramsey Reset test, χ^2^ normality is for Jargue-Bera test of normality, and χ^2^ LM for Breusch-Godfrey serial correlation LM test. *p* values of diagnostic tests are in parenthesis. ***p < 0.01, and **p < 0.05

The relationship between economic instability and GHG emissions is found to be significant and positive, such that that a 1% increase in economic instability results in an increase of 0.099% of GHG emissions in the long-run. The results indicate that as economic instability increases, GHG emissions also rise in the Netherlands. This finding is in line with previous research by Ozili [75], which suggests that economic instability can lead to increased emissions due to the financial decisions made during such periods. Banks and financial institutions play a critical role in this dynamic, as their lending practices can influence corporate behavior. During times of economic instability, banks may be more inclined to finance high-emission sectors or industries that are less environmentally sustainable, exacerbating the environmental impact [66, 100, 103, 104]). This relationship highlights the need for financial institutions to integrate climate risk into their decision-making processes, prioritizing investments that support low-carbon initiatives and environmentally responsible practices [22, 52, 101]. By doing so, banks can contribute positively to the environment, even in the face of economic challenges.

The study also reveals a positive relationship between industrialization and GHG emissions, such that a 1% increase in industrialization results in an increase of 0.517% of GHG emissions in the long-run, with industrialization identified as a significant contributor to environmental degradation in the Netherlands. This finding is consistent with previous studies by Raihan et al. [81], and Sikder et al. [87], which demonstrate the adverse environmental effects of industrial activities, particularly those reliant on fossil fuels for energy production [56, 93]. Industrialization, while driving economic growth, also leads to increased GHG emissions due to the energy-intensive nature of manufacturing processes, transportation, and the use of construction materials like cement [61, 62, 100]. The Dutch government’s National Climate Agreement, which targets a 95% reduction in GHG emissions by 2050, underscores the urgency of mitigating the environmental impact of industrialization [64, 91]. While efforts are being made to reduce emissions through technological advancements and regulatory measures, the study emphasizes the importance of balancing industrial growth with environmental sustainability.

**Fig. 5:**
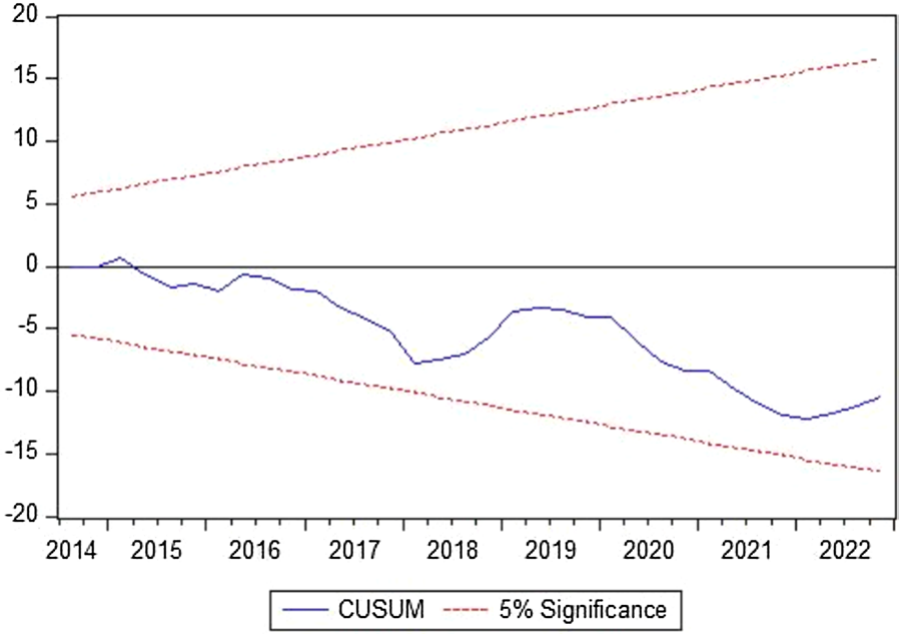
The cumulative sum of the recursive residual plot

**Fig. 6:**
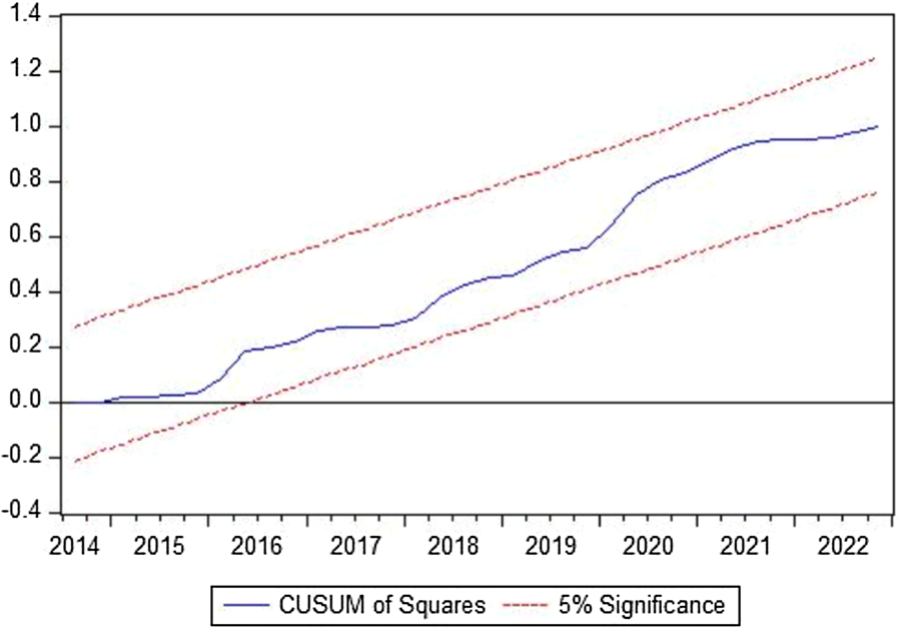
The cumulative sum of the square of the recursive residual plot

The ARDL short-run analysis further supports the findings, showing that the circular economy, technological innovation, and environmental taxes have a mitigating effect on GHG emissions in the Netherlands. In contrast, economic instability and industrialization are associated with higher emissions. The estimated error correction model coefficient indicates that any short-term deviations from the long-run equilibrium are quickly corrected, reinforcing the stability of the relationships observed. Diagnostic tests confirm the robustness of the model, and the stability plots suggest that the results are reliable. Figures 5 and 6 display the stability of the models at a 5% significance level through the CUSUM and CUSUMQ plots. These findings have significant implications for policy-making, emphasizing the need for continued support of circular economy practices, technological innovation, and environmental taxes to achieve long-term sustainability goals.

### Robustness check

#### FMOLS and DOLS

This study employs FMOLS and DOLS techniques to validate the robustness of the long-run estimates obtained from the ARDL model. Both FMOLS and DOLS are sophisticated methods designed to address potential endogeneity and serial correlation issues in time series analysis. The consistency of the results from FMOLS and DOLS with those obtained from the ARDL model, as presented in Table 6, confirms the reliability of the long-run relationships identified between GHG emissions and various explanatory variables. This alignment underscores that the observed relationships are robust and not artifacts of econometric concerns.

**Table 6:** Robustness check

Dependent variable: lnGHG emissions	FMOLS			DOLS		
	Coefficient	t-statistics	Prob	Coefficient	t-statistics	Prob
lnCircular economy	−0.667***	−2.999	0.004	−0.619***	−3.364	0.002
lnTechnological innovation	−0.404***	−3.747	0.000	−0.660***	−3.801	0.000
lnEnvironmental tax	−0.785***	−7.926	0.000	−0.951***	−5.763	0.000
lnEconomic instability	0.176*	2.172	0.088	0.208*	2.185	0.065
lnIndustrialization	0.558***	3.254	0.002	0.428***	3.664	0.000
Constant	2.804***	3.439	0.000	2.638***	3.793	0.000
R^2^	0.910			0.976		
Adjusted R^2^	0.900			0.958		

***p < 0.01, **p < 0.05, and *p < 0.10

The robustness analysis affirms that promoting circular economy practices, technological innovation, and the environmental tax effectively reduces GHG emissions in the Netherlands, while economic instability and industrialization are positively associated with increased GHG emissions [97]. These findings support the use of these strategies in policy and decision-making, ensuring that efforts to mitigate GHG emissions are grounded in reliable and consistent evidence. By reinforcing the stability of the ARDL results, the robustness analysis highlights the importance of integrating environmental sustainability into economic and industrial policies to achieve meaningful and enduring reductions in GHG emissions.

#### Wavelet coherence analysis

The Wavelet Coherence Analysis (WCA) provides a comprehensive view of the interactions between GHG emissions and several explanatory variables, including circular economy, technological innovation, environmental tax, economic instability, and industrialization, over the period from the first quarter of 2010 to the fourth quarter of 2022. The gray cone in the WCA visualizations represents the area of effect, while the thick black line, derived from Monte Carlo simulations, denotes the significance level. This approach captures both the causation and correlation in the relationships, offering insights into how these dynamics evolve across different time scales and frequencies [58].

**Fig. 7:**
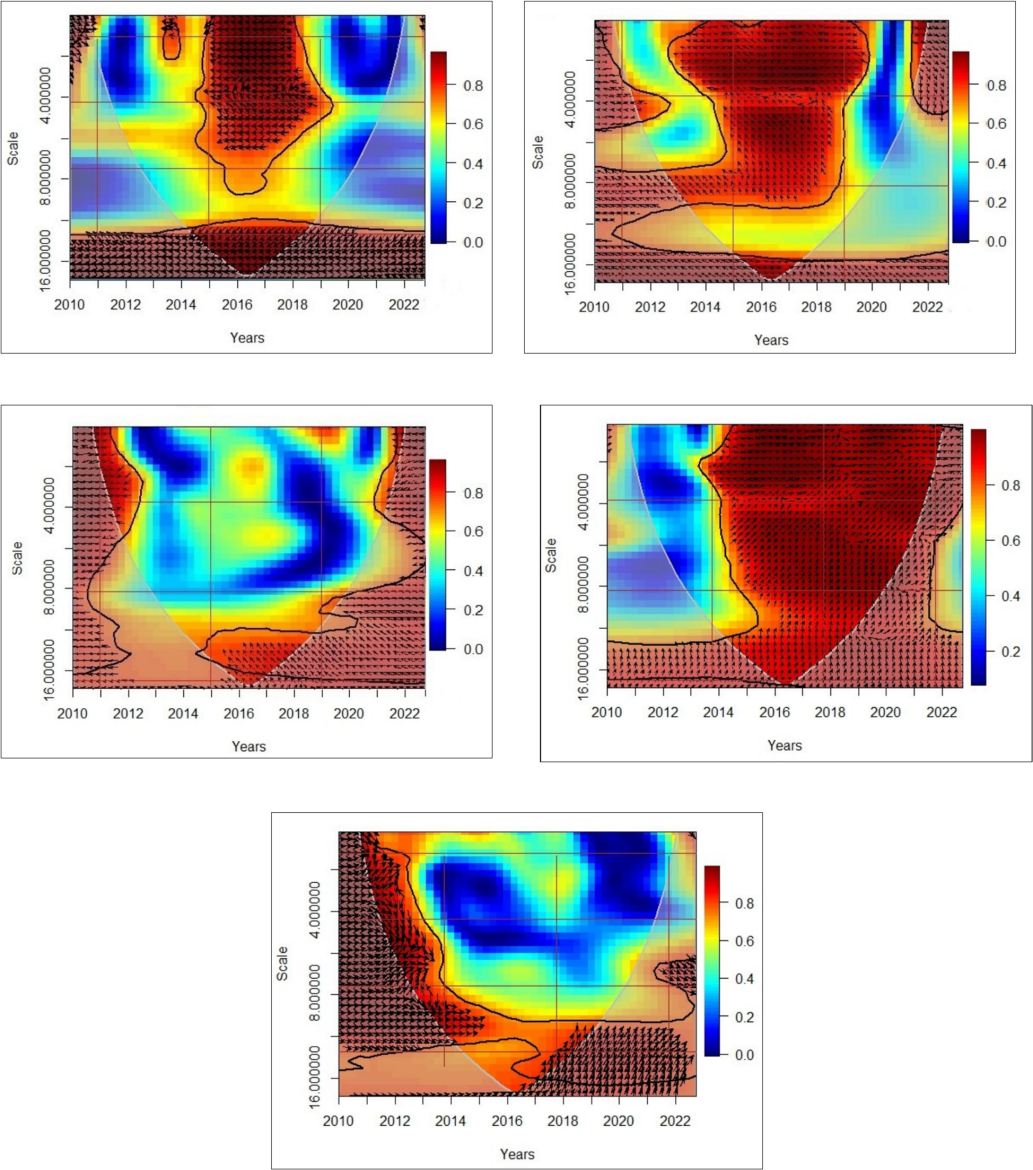
Wavelet coherence analysis. Time and frequency are presented on the horizontal and the vertical axis, respectively. On the wavelet coherence plots, the black contour shows the 5% significance level, and regions with strong co-movements are represented by warmer colors (*red*), whereas colder colors (*blue*) represent regions with weak co-movements. The *arrows* provide the direction of interdependence and causality relationships. *Arrows* pointing to the *right* (→) indicate that variables are positively correlated. *Arrows* pointing to the *left* (←) indicate that variables are negatively correlated. The ↗ and ↙ *arrows* mean that first variable leads second variable, whereas the ↘ and ↖ *arrows* indicate that the second variable leads the first variable. The *straight up* (↑) and *down* (↓) *arrows* imply that the first variable is leading and lagging, respectively. **a** WCA between GHG emissions and circular economy. **b** WCA between GHG emissions and technological innovation. **c** WCA between GHG emissions and environmental tax. **d** WCA between GHG emissions and economic instability. **e** WCA between GHG emissions and industrialization

Figures 7a–e illustrate the findings from the WCA. The findings illustrated in Fig. 7a indicate a noticeable correlation between the circular economy and GHG emissions, especially during the period from 2014Q2 to 2019Q2. The prevalent upward and leftward arrows across various frequencies suggest that the adoption of circular economy practices is associated with a decrease in GHG emissions. Circular economy principles emphasize resource efficiency, waste reduction, and the reuse and recycling of materials, resulting in lower emissions by minimizing the demand for raw materials, reducing energy consumption, and decreasing emissions from waste processing [104]. From 2014Q2 to 2019Q2, the relationship became more evident, suggesting that the increased adoption of circular economy practices corresponded with a significant reduction in GHG emissions. This trend indicates that the circular economy is effective in reducing emissions and serves as a reliable predictor of fluctuations in emissions. The data indicates that the circular economy can predict substantial changes in GHG emissions in the Netherlands, underscoring its potential as an effective mechanism for reducing environmental impact.

Figure 7b illustrates a strong negative correlation between technological innovation and GHG emissions, indicating that technological advancements significantly contribute to GHG emissions reduction over time. The persistent upward and leftward trends across various frequencies from 2010Q1 to 2019Q3, along with the sustained negative correlation at low frequency between 2019Q4 and 2021Q2, indicate a direct relationship between technological innovations and reduced emissions. Technological innovations, including the advancement of cleaner energy sources, enhanced industrial processes, and optimized resource management technologies, mitigate the environmental impact of production and consumption. The adoption of advanced technologies enables industries to decrease energy consumption, reduce waste, and minimize carbon emissions. The persistent occurrence of these negative arrows supports the notion that technological advancement is a dependable indicator of emissions reductions, aiding in the prediction of substantial decreases in GHG emissions in the Netherlands throughout the examined timeframe.

Figure 7c illustrates a negative correlation between GHG emissions and environmental tax, underscoring the efficacy of environmental tax policies in mitigating GHG emissions over time. The leftward-up arrows across various scales (frequencies) indicate that an increase in environmental tax correlates with a decrease in GHG emissions, demonstrating a clear inverse relationship. Environmental taxes increase the cost associated with carbon-intensive activities, thereby incentivizing businesses to adopt sustainable practices, invest in cleaner technologies, and reduce their carbon footprint. This policy mechanism promotes energy efficiency and facilitates a transition to greener alternatives, resulting in reduced emissions. Between 2010Q1 and 2022Q4, a consistent negative relationship was found between environmental taxes and GHG emissions. This supports the idea that these taxes are a reliable way to predict decreases in GHG emissions in the Netherlands, as businesses and consumers change their behavior in response to the financial incentives created by these policies.

Figure 7d indicates a robust positive correlation between economic instability and GHG emissions, as evidenced by the consistent presence of rightward-up arrows across multiple scales from 2013Q4 to 2022Q4. This suggests that economic instability correlates with heightened GHG emissions. Economic instability frequently results in unpredictable fluctuations in production levels, alterations in energy consumption patterns, and a possible dependence on more polluting energy sources as industries endeavor to sustain stability. Moreover, during periods of economic distress, there is often an increase in resource consumption and a decline in efficiency, which further intensifies emissions. Between the first quarter of 2010 and the third quarter of 2013, the relationship is predominantly observed at high scales (low frequency), indicating that long-term fluctuations in economic instability exert a more substantial influence on GHG emissions during this timeframe. The data indicates that economic instability serves as a reliable predictor of heightened GHG emissions in the Netherlands, illustrating how it can lead to significant alterations in emission levels through changes in industrial activity, energy consumption, and regulatory responses.

The results in Fig. 7e illustrate a clear positive relationship between industrialization and GHG emissions over the period from 2010Q1 to 2022Q4, as indicated by the predominance of rightward and upward arrows at various scales. From 2010Q1 to 2014Q1, this correlation is visible across low, medium, and high-frequency scales, suggesting that industrialization consistently drives an increase in GHG emissions across different time frames. This is likely due to increased energy consumption, higher production outputs, and greater resource utilization associated with industrial processes. From 2014Q2 to 2022Q4, the association is particularly strong at high and intermediate scales (low and middle frequencies), which suggests that as industrialization progresses, it continues to be a dominant factor in the rise of GHG emissions, reinforcing the idea that industrial expansion leads to greater emissions. Thus, the unfolding of industrialization in the Netherlands serves as a reliable predictor of significant fluctuations in GHG emissions during this period.

#### Causality tests

To validate the findings from the Wavelet Coherence Analysis, this study utilized the Gradual Shift Causality (GSC) and Toda-Yamamoto (TYC) tests, revealing distinct causal relationships between GHG emissions and various factors. The results demonstrate a bidirectional causality between GHG emissions and the circular economy, indicating that the circular economy both impacts and is influenced by GHG levels. This aligns with prior studies, which emphasize the reciprocal influence between these variables. In contrast, a unidirectional causality is observed from technological innovation, environmental tax, economic instability, and industrialization to GHG emissions. Technological innovation reduces emissions, environmental taxes provide economic incentives for emission reductions, economic instability affects emission levels, and industrialization increases emissions. These findings are consistent with existing literature and underscore the need for integrated strategies that address both predictive and reactive aspects of GHG emissions management (Table 7).

**Table 7:** Causality results

Causality path	Gradual shift causality test		Toda-yamamoto causality test	
	Mwald	*p*-value	Wald	*p*-value
GHG emissions → Circular economy	19.231*	0.000	19.897*	0.000
Circular economy → GHG emissions	20.312*	0.00	21.036*	0.000
GHG emissions → Technological innovation	3.787	0.544	2.998	0.636
Technological innovation → GHG emissions	18.676*	0.003	19.687*	0.000
GHG emissions → Environmental tax	2.782	0.502	1.778	0.628
Environmental tax → GHG emissions	9.983*	0.027	10.019*	0.023
GHG emissions → Economic instability	1.782	0.318	2.136	0.309
Economic instability → GHG emissions	16.527*	0.009	17.390*	0.007
GHG emissions → Industrialization	3.672	0.539	3.905	0.532
Industralization → GHG emissions	18.562*	0.004	17.951*	0.006

* indicates the presence of causality

## Conclusions and policy implications

### Concluding remarks

The Netherlands has committed to the European Union’s ambitious targets of reducing GHG emissions by 2030 and achieving carbon neutrality by 2050. To meet these goals, the Dutch government has introduced a range of policy initiatives focused on sustainability and the circular economy. Central to this effort is the aim to transition to a circular economy by 2050, which involves utilizing sustainable and renewable materials, designing products for durability and recyclability, and minimizing waste through practices such as reuse, repair, and refurbishment. The circular economy plays a crucial role in reducing GHG emissions by enhancing the efficiency of material flows and extending the lifespan of products, thus reducing waste. Despite these efforts, a significant challenge remains: the Netherlands consumes approximately 221 million tons of materials annually, with 167 million tons not being reintegrated into the economy, underscoring the need for more effective circular practices.

In examining the long-term and causal effects of the circular economy, technological innovation, environmental tax, economic instability, and industrialization on GHG emissions from 2010Q1 to 2022Q4, this study employed ARDL bounds testing methods to analyze these relationships. The co-integration tests confirmed that GHG emissions, the circular economy, technological innovation, environmental tax, economic instability, and industrialization are interrelated. The ARDL analysis found that the circular economy, technological innovation, and environmental tax have a negative association with GHG emissions, whereas economic instability and industrialization contribute positively to emissions. The finding highlights the significant impact of the circular economy in reducing GHG emissions through improved material flows and decreased reliance on fossil fuels and non-metallic minerals.

### Policy implications

To advance these findings and facilitate sustainable development, the study recommends several policy measures for Dutch decision-makers. Enhancing construction practices to focus on material reuse and renovation, implementing a circular food system with local production and waste utilization, and transitioning from fossil fuels to renewable energy sources are critical steps. Additionally, boosting the recycling and high-value recycling of imported materials and promoting repair and remanufacturing processes will be vital. Transforming cities into exemplars of the circular economy can drive behavioral and business changes regionally and nationally. Embracing risks and learning from setbacks will be essential for success. Local governments should lead by example, adopting sustainable products and circular infrastructure practices, such as recycled materials for roads and buildings, to position themselves as leaders in the transition to a circular economy and reduce reliance on fossil fuel extraction.

1. In line with the European Green Deal and the Netherlands’ Climate Agreement, policies should encourage sustainable practices in important industries in order to lower GHG emissions and strengthen the country’s circular economy. Stronger implementation of carbon pricing mechanisms, such as a more robust carbon tax or an expansion of the EU Emissions Trading System (ETS) to encompass more industries, are among the specific proposals. By supporting technological innovation in recycling, encouraging expanded producer responsibility, and offering tax incentives or subsidies to companies that embrace circular models, the government may help promote the circular economy. Furthermore, strengthening public–private partnerships to promote cooperation on waste reduction and green technology will support the EU’s goals of reaching net-zero emissions by 2050. These policies will significantly reduce emissions while promoting economic growth through the circular economy, especially when combined with initiatives from the Netherlands’ Climate Agreement.
2. To guarantee that manufacturers assume accountability for the whole lifespan of their goods, promoting eco-friendly design and cutting waste, strengthen and broaden extended producer responsibility programs. Create required product information systems that reveal environmental effects and provide financial rewards to companies that embrace eco-design concepts (e.g., recyclability, durability, and the use of sustainable materials).
3. To promote sustainable production and consumption, provide financial incentives (such as grants or tax breaks) to companies using circular models like leasing, remanufacturing, and sharing. To lessen dependency on virgin resources, make investments in updating recycling systems, guaranteeing effective waste separation and collection, and providing incentives for the use of recycled materials in manufacturing processes.
4. To encourage the adoption of circular processes and lower carbon footprints, strengthen carbon pricing systems (such as carbon taxes) and set emission reduction objectives for industries. Include circular economy standards in public procurement guidelines, giving sustainable, recyclable, and repairable goods first priority when it comes to government purchases. This will increase demand for circular solutions.

### Research limitations and future research directions

This study is constrained by its sole emphasis on the Netherlands, and future research might benefit from using panel data to improve the generalizability and robustness of the findings across several nations or regions. Furthermore, while this study used ARDL, FMOLS, DOLS, and the wavelet coherence analysis, future studies might use more sophisticated approaches to reflect the complex and dynamic nature of energy diversification. Machine learning, for example, has the potential to find non-linear correlations between variables and enhance prediction accuracy. Structural Equation Modeling (SEM) might be used to investigate complex interdependencies across various elements, whereas Granger Causality with Time-Varying Parameters would provide a better understanding of causal links over time, adjusting to changes in economic and policy contexts.

Future research would benefit from broadening the scope of the study to include variables such as natural capital, structural changes, and government governance. Furthermore, it would be interesting to investigate the moderating or mediating role of these variables in the link between the circular economy and GHG emissions. These elements may have a substantial impact on the circular economy and give a more complete knowledge of the dynamics that shape circular economy systems. Furthermore, future research should look at whether including these factors changes the current study’s findings, thereby refining policy recommendations and improving the accuracy of circular economy models.

## Declaration of generative AI in scientific writing

During the preparation of this work the author(s) used ChatGPT in order to improve the readability and language of the manuscript. After using this tool/service, the author(s) reviewed and edited the content as needed and take(s) full responsibility for the content of the published article.

## Data Availability

The data used in this study is openly available on the official websites of "World Development Indicators" and "Eurostat".

## Footnotes

1 NEDERLAND FIT FOR 55? Mogelijke gevolgen van het voorgestelde EU-klimaatbeleid (https://open.overheid.nl/documenten/ronl-29514ee5-ee92-44f6-aec0-4910c6afb6f6/pdf) (In Dutch language). Accessed on 15 March 2025.
